# Abiotic factors influencing the health of mature *Araucaria heterophylla* (Norfolk Island Pine trees) in urban coastal parks

**DOI:** 10.1007/s00267-026-02471-8

**Published:** 2026-04-21

**Authors:** Anna Petrova, Chris Pratt, Ruby Naomi Michael

**Affiliations:** 1 Green Infrastructure Research Labs (GIRLS), Griffith Institute for Human and Environmental Resilience, Griffith University, Griffith, QLD Australia; 2 School of Environment and Science/Australian Rivers Institute, Griffith University, Griffith, QLD Australia; 3 School of Engineering and Built Environment, Griffith University, Griffith, QLD Australia

**Keywords:** Urban soil, Tree management, Tree decline, Nutrients, Urban infrastructure

## Abstract

Tree decline can significantly impact both the aesthetic value and ecological function of urban forests. Understanding the abiotic factors influencing tree health is essential for developing effective management strategies. This study investigated the decline of heritage *Araucaria heterophylla* (Norfolk Island Pine) in urban areas of the Gold Coast, Queensland, Australia, with a focus on identifying underlying abiotic causes. General assessment was conducted along a 5-km coastal strip (including 946 trees); climate conditions on the Gold Coast and Norfolk Island were compared; and 38 selected trees of varying health status were examined in detail. This included soil physicochemical and nutrient analysis, as well as foliar nutrient profiling. Results showed that 15.8% of the 946 initially assessed trees exhibited symptoms of decline, such as dieback, browning of foliage tips, extensive defoliation and shortened foliage length. Mean temperatures on the Gold Coast were consistently higher than those in the species’ native range and the current decline followed a period of elevated temperatures and reduced rainfall. Declining trees (*n* = 32) from the subset population were found to be growing in compacted soils, with significantly elevated foliar sodium and lower foliar carbon levels compared to healthy trees (*n* = 6), indicating possible disruption of nutrient regulation. Half of the trees in the advanced stage of decline were located less than 5 m away from carparks (50%), which was higher when compared with Moderate Decline (18.7%) and very healthy Benchmarking trees (0%). Most trees in the advanced decline category were not mulched and had turfgrass as the primary groundcover (62.5%), whereas trees in moderate decline were more commonly associated with turfgrass combined with fallen branchlets (43.75%). In addition, all Benchmarking trees were located in sandy soils, whereas declining trees were more commonly associated with loam soils, which are more susceptible to compaction. Key abiotic stressors identified were extreme weather and proximity to urban infrastructure. Management recommendations include improving root zone conditions by providing irrigation during dry periods, mulching, implementing routine health monitoring to support early intervention and, for new planting, increasing distances from hard infrastructure.

## Introduction

Urban trees are recognized for their benefits to physical and mental health, their role in supporting urban wildlife and their ability to reduce temperatures by providing shade, creating a more comfortable environment for residents. However, urban tree health is increasingly compromised by abiotic stressors such as extreme weather events, reduced soil moisture, soil quality and compaction and disturbance from frequent construction activities (Day and Bassuk, 1994; Lu et al., 2018; Scharenbroch et al., 2005; Yang et al., 2016).

Urban plantings often consist of a mix of native and exotic species selected for their ornamental value, site suitability and maintenance considerations (Roy et al., 2017). *Araucaria heterophylla* (Salisb.) Franco, commonly known as the Norfolk Island Pine tree, is native to Norfolk Island, which is a remote external territory of Australia. It was introduced to many countries at various times and to the Australian mainland specifically in the late 18^th^ century, where it is now considered well-adapted to the local environment. Since its introduction, this species has been widely planted across most Australian states, including Queensland, particularly along the coast and now constitutes a substantial proportion of its urban forest. Some of these trees, now over a century old, are recognized for their national significance (National Trust of Australia, 2025).

There are several physiological traits that enable Norfolk Pine trees to grow in the Australian coastal conditions. *Araucaria heterophylla* is an extremely isohydric tree, maintaining its daily minimum leaf water potential constant regardless of its soil water potential (Zimmer et al., 2016). *Araucaria heterophylla* is also a halophyte, capable of surviving in environments with high salt concentrations in the rhizosphere. Although no studies have specifically investigated the mechanisms of sodium (Na⁺) exclusion in *A. heterophylla*, higher plants are generally known to employ several strategies to manage Na⁺. These include controlling Na⁺ uptake and loading into the xylem, retrieving Na⁺ from the xylem, extruding it from roots, compartmentalising it into vacuoles within cells and excreting it through salt glands or roots (Zhang et al., 2010).

While isohydricity and halophytic traits are valuable for survival, external environmental factors, including heatwaves, topography and soil characteristics, can override physiological drought resistance (Feng et al., 2019). Dieback events of this species have been documented since the mid-20th century: reported symptoms include top dieback, browning of foliage tips, chlorotic spots, defoliation and shortened foliage length. Previous research in Australia has linked these symptoms to various causes, including elevated sodium levels influenced by surfactants (Dowden and Lambert, 1979; Grieve and Pitman, 1978; Moodie et al., 1986) and the impact of pathogens (Darge, 2017; Golzar and Burgess, 2011; Huang and Wang, 2011). Anecdotal accounts also suggest that native bird species, such as corellas and rainbow lorikeets, can cause substantial physical damage to the trees in some locations (*Multiple local residents, personal communication, March 2023*). The tree decline was also observed in other countries and in the native habitat on Norfolk Island and linked to pathogens (Darge, 2017; Gupta et al., 2010; Phytophthora dieback on Norfolk Island: Diagnosis and management, 2022; Terhem et al., 2021) and intense competition from non-native grasses and shrubs, combined with overgrazing and soil nutrient depletion on land previously cleared for agriculture (Benson, 1980).

In the Gold Coast region, Australia, where these trees are widespread, evidence suggests that *A. heterophylla* has long exhibited variable performance. Historic photographs in the local electronic catalogue (Gold Coast Libraries, n.d.) and from local Facebook groups such as ‘Old Gold Coast’ (n.d.) and ‘Gold Coast Historical Society and Museum’ (n.d.) demonstrate that tree dieback has been occurring throughout the last century. While some individuals have thrived for decades, others experience stunted growth or sudden decline after reaching a certain age.

This study aimed to investigate the recent decline of *A. heterophylla* in urban coastal areas of the Gold Coast by identifying key abiotic stressors and assessing their influence on tree health, with the goal of informing future urban tree management. Specifically, the objectives were to (i) characterise the environments of trees exhibiting different health conditions in coastal urban parks, including soil physicochemical properties and proximity to built infrastructure and (ii) determine whether these factors are associated with changes in physicochemical properties of trees’ foliage and with trees’ health overall. It was hypothesised that soil conditions and urban infrastructure are significant contributors to the observed decline.

## Materials and Methods

### Regional Climate And Weather Context

The Gold Coast region of South East Queensland, Australia, experiences higher mean annual rainfall and temperatures compared to the native range of *A. heterophylla* on Norfolk Island. For example, mean annual rainfall at Coolangatta (weather station 040717) is 1517 mm, while on Norfolk Island (weather station 200288) it is 1282 mm. Average monthly temperatures on the Gold Coast range from 20.7 °C in July to 28.5 °C in January, compared to a range of lower temperatures - 18.4 °C to 25.0 °C - on Norfolk Island (BoM, 2025). Temperature extremes on the Gold Coast are considerably higher, with recorded summer maximums reaching 40 °C, whereas the historical maximum on Norfolk Island is 28.5 °C (BoM, 2025).

In the years preceding this study, the Gold Coast experienced a combination of reduced rainfall and elevated temperatures. 2019 was one of the driest years on record, with an annual rainfall total of 825.6 mm - only marginally above the 1986 record low of 792.6 mm. Rainfall in 2018 was also below the annual average, at 1046.2 mm. Another very dry year in the 21^st^ century was 2013, with 994.2 mm.

Prior to the dieback event, peak daily summer temperatures exceeded the monthly long-term means of 28.5 °C in January and 27.5 °C in December. In 2019, they reached 30.1 °C and 29.2 °C, respectively. The annual mean temperatures were also higher than the climatological norm: 25.9 °C in 2017 and 2019 (BOM, 2025).

### Urban Forest Assessment

**Fig. 1:**
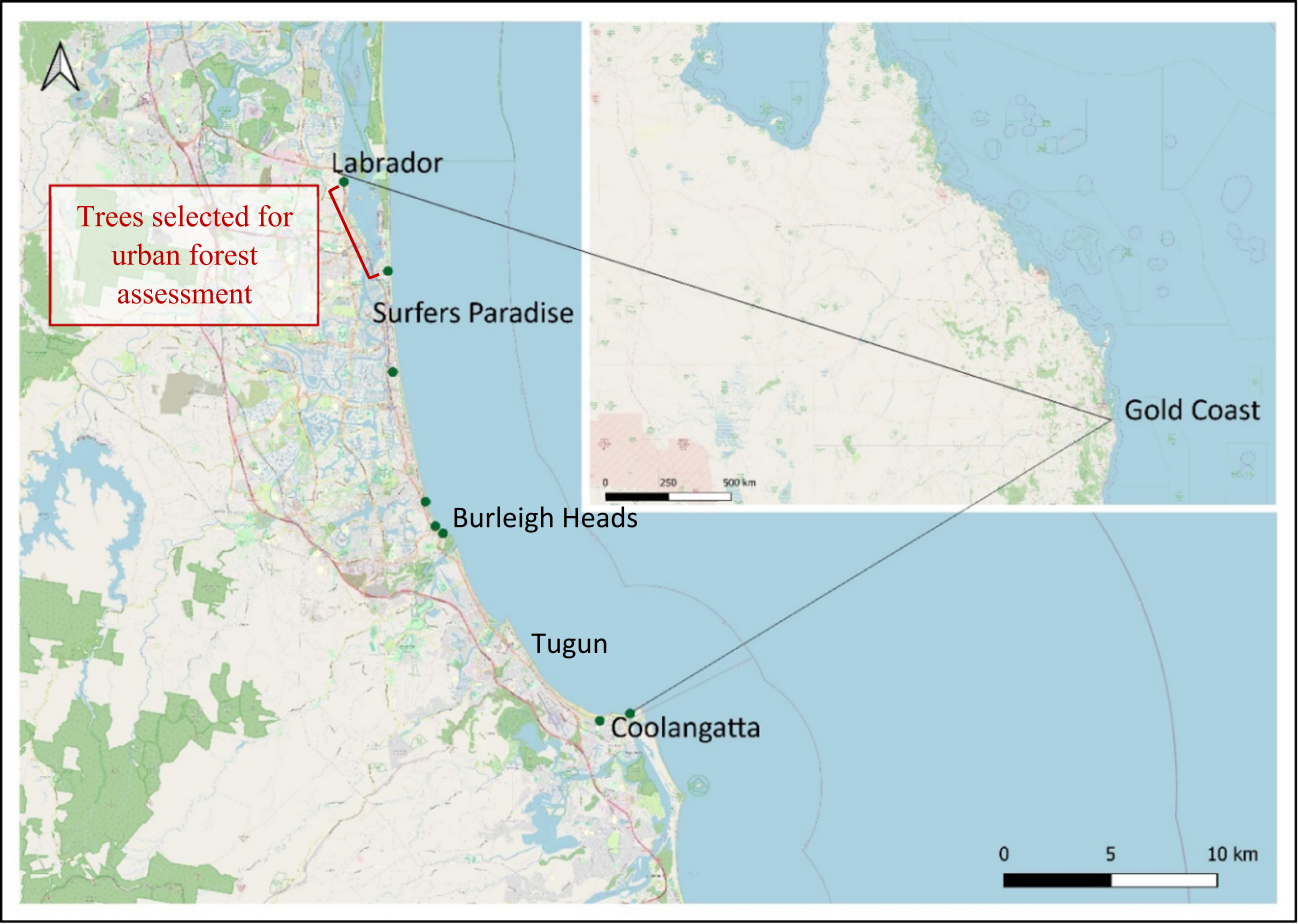
Eight parkland study sites are shown as green dots located along the Gold Coast in Queensland, Australia, between the suburbs of Labrador and Coolangatta. Additionally, six benchmark trees were located in Burleigh Heads and Tugun. The map was generated with QGIS

To understand how widespread the decline is, a visual assessment of *A. heterophylla* in urban parks located along the shoreline of the Gold Coast was conducted in November—Australian spring. This assessment aimed to gauge the scale of the issue rather than investigate the causes of dieback and evaluated trees along a 5 km stretch from Labrador to Surfers Paradise (Fig. 1). The field assessment used a modified version of Roman et al. (2020) tree assessment protocol: their percentage of canopy dieback was estimated, visible from the ground dead branches were counted, change in the pyramidal shape and foliage discolouration were noted and photographs were taken. The visual assessment identified trees with more than 30% dieback due to defoliation, resulting in the loss of the typical *A. heterophylla* pyramidal shape. All assessed healthy and declining trees located in foreshore parks were counted and their percentage distribution across two categories - i.e., >30% dieback and <30% dieback - was calculated (Table 1).

**Table 1:** Categorisation of tree health based on the canopy dieback, branch loss and foliage discolouration

Canopy Dieback (%)	Visual Indicators	Tree health category	Percentage of trees in each category (%)	
0–5%	Canopy symmetrical; no dead branches, very minimal or no tips browning of branchlets^a^, green foliage	Benchmarking	5	
6–10%	Minor browning or branchlets loss, ≤1–2 dead branches, crown shape intact, discolouration of branchlets’ tips	Moderate Decline I	5	
11–30%	>2 dead limbs, crown discolouration and/or thinning, discolouration of branchlets’ tips	Moderate Decline II	20	
31–50%	Noticeable thinning, multiple dead branches, reduced number of branchlets density towards the trunk; distortion in natural silhouette, discolouration of branchlets’ tips	Advanced Decline I	20	
51–75%	Sparse foliage; extensive dieback of primary limbs; leader or top crown dead, discolouration of branchlets’ tips	Advanced Decline II	25	
>75%	Near-total or total loss of foliage; mostly dead limbs; severe structural risk	Advanced Decline III	25	

^a^Small distal subdivision of primary lateral branches

### Tree Selection

Eight parks were pre-selected by a consultant from Heritage Tree Care, a council contractor responsible for soil data collection. Site selection aimed to include two trees exhibiting advanced decline and two exhibiting moderate decline within each park, with locations distributed along Queensland’s Gold Coast coastal gradient, between Labrador and Coolangatta. An equal number of *A. heterophylla* in advanced (*n* = 16) and moderate (*n* = 16) stages of decline growing between 20 and 100 m away from the shoreline was selected for sampling. Each location had four trees—two in Moderate and two in Advanced Decline. Although the exact age of the trees could not be determined due to limited data, their trunk diameter suggests the selected trees were approximately 37 to 100 years old.

Due to the lack of a comprehensive guideline for benchmarking, an additional six healthy trees with no more than 5% dieback were included to provide a benchmark. This relatively small figure reflects the limited availability of very healthy trees on the Gold Coast. All six trees were also located along the shoreline, in Burleigh Heads and Tugun (Fig. 1).

**Fig. 2:**
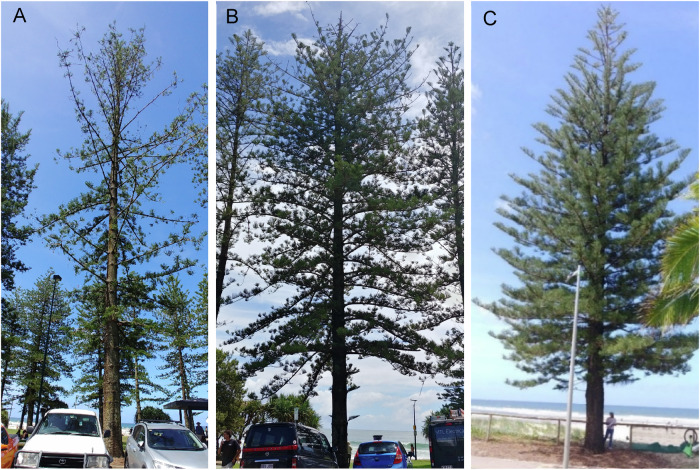
Representatives of three levels of decline of *Araucaria heterophylla* - **A** Advanced Decline, **B** Moderate Decline and **C** Benchmarking

Trees were assessed by the primary author and an experienced, qualified arboriculture specialist based on visual parameters, including canopy density, the percentage of dieback, canopy colour and foliar damage. While health increments were between 5% and 25% and decline varied in severity, which was reflected in classes I, II and III (Table 1), for the analysis, the trees were separated into two categories: Advanced Decline (AD) with >31% dieback and Moderate Decline (MD) with 6-30% dieback. The Benchmarking (B) trees had ≤5% of dieback (Fig. 2A, B, C, respectively).

**Fig. 3:**
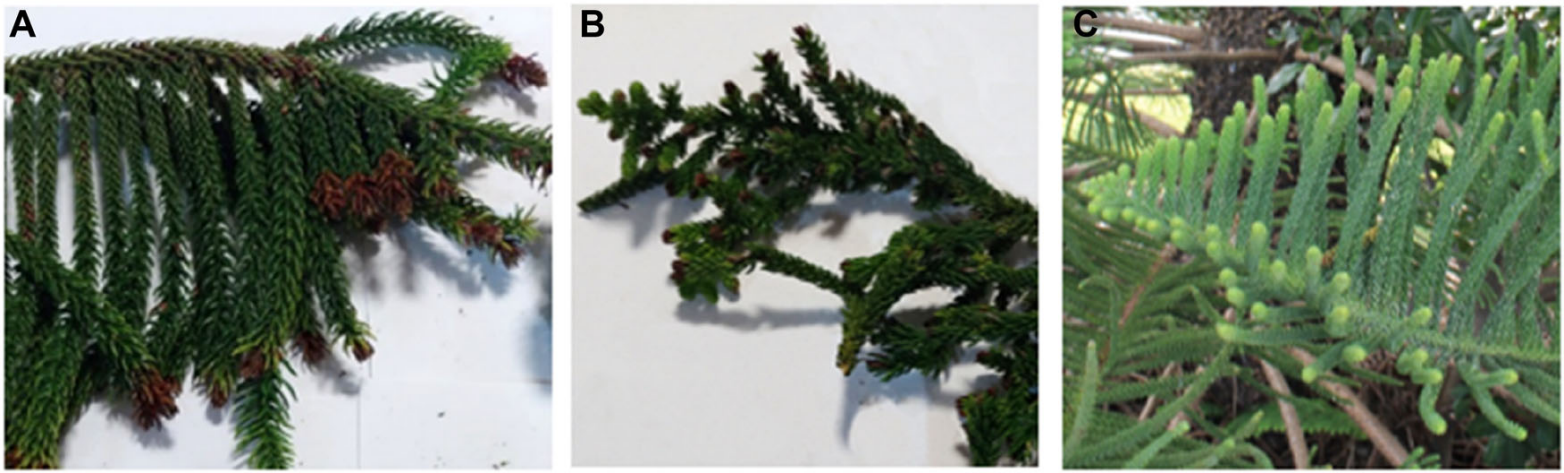
Examples of the foliage of the three groups of *Araucaria heterophylla*—**A** Moderate Decline, **B** Advanced Decline and **C** Benchmarking

**Fig. 4:**
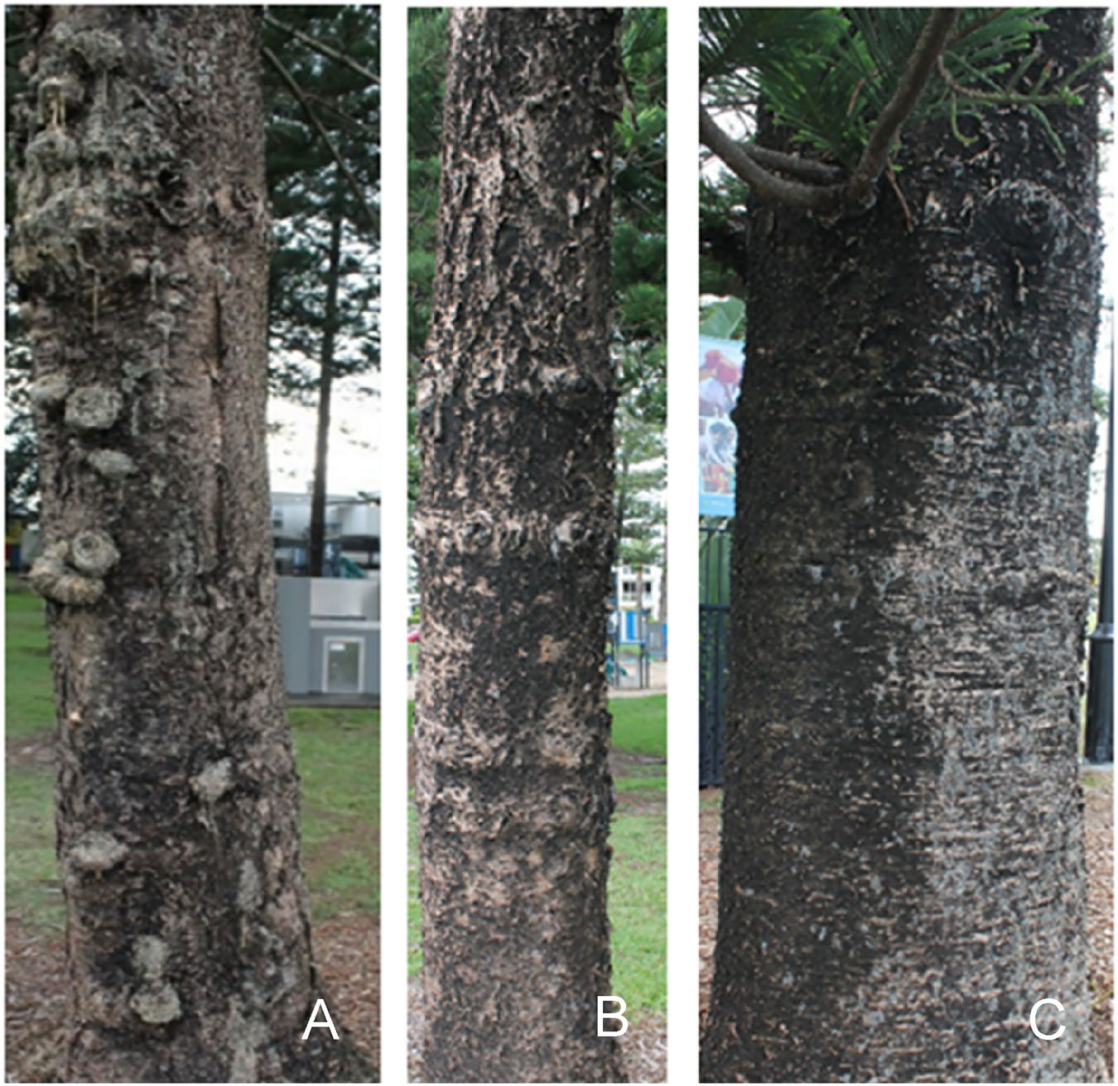
Examples of trunks of **A** Advanced Decline, **B** Moderate Decline and **C** Benchmarking trees

Tree height, diameter at breast height (DBH) and the average foliage length were also recorded. The trees were inspected from four directions for signs of damage (Fig. 2), leaf damage (Fig. 3) and the presence of resin, moss, lichen and fungus (Fig. 4).

### Groundcover

The trees’ groundcover was assessed and seven groups were identified—turfgrass only, a combination of bare ground and mulch at ≤1 m radius, a combination of grass and mulch at ≤1 m radius, mulch mixed with fallen branchlets at ≤1 m radius, grass with fallen branchlets, a combination of grass, bare ground and fallen branchlets and mulch to dripline with fallen branchlets. The percentage of trees in the identified categories was calculated.

### Temperature Measurements and Proximity to Infrastructure

**Fig. 5:**
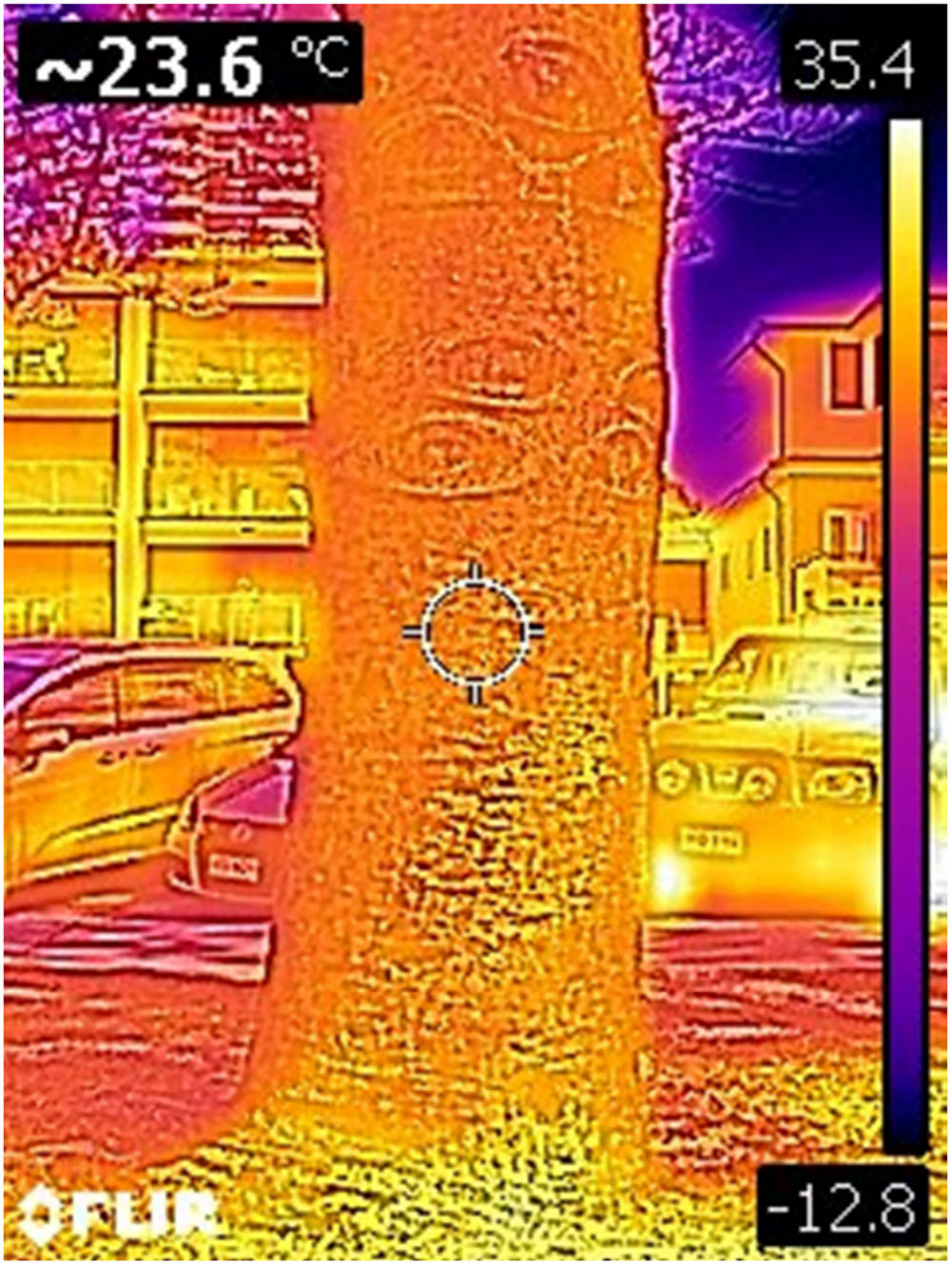
An example of a thermal image taken with a Vevor camera. The temperature of the tree surface in the central circle is shown in the top left corner

Tree trunk surface temperature was measured in a random order to avoid bias using a Vevor thermal camera during the Australian spring (mid-November), between 10:00 and 11:00 am, over two sampling days. The maximum air temperatures on these days were 27.6 °C and 27.2 °C. The camera was oriented westward and aimed at the central portion of each tree trunk at a height of 1.5 m and from a distance of 3 m (Fig. 5). The interval between thermal images of Advanced Decline and Moderate Decline trees within each location ranged from 1 to 10 min. Since the six trees selected for benchmarking were spread too far apart along the coast, which greatly affected the time of day for temperature reading, they were not included in this analysis.

The surrounding landscape of each tree was assessed, noting four types of infrastructure: (1) car parks, (2) roads, (3) footpaths and (4) other built infrastructure. The ‘other’ category included toilet blocks, built shade structures, communal barbecues, playgrounds and clearly observable underground infrastructure. Distances to each type of infrastructure were measured and recorded; features located at a distance of 5 m or greater were classified as $\ge \,$ m.

### Soil and Foliage Sampling

Soil of the sampled area characterised as rudosols (Queensland Globe, 2026)—young, least developed soils with low fertility (Bryant et al., 2012). It was sampled by the contractor prior to the beginning of the study, 1 m away from the tree trunk with an auger, targeting the top 30 cm of the soil profile. Prior to sampling, the top mulch or grassy layer was removed. Soil cores (15 cm diameter; approximately 5.3 L volume) were extracted and homogenised. A subsample of 300 g from each core was placed in zip-lock bags, stored on ice in the field and subsequently kept at 4 °C in the laboratory until processing, which occurred within one week of collection.

For foliage nutrient analysis, between 15 and 20 branchlets with no visible new growth were collected in autumn from lower, mature branches, yielding approximately 200–300 g of leaves per tree. Samples were placed in paper bags and stored in a cool box in the field prior to further processing (Rautio and Fürst, 2013). The length of three branchlets randomly selected from each sample was measured.

The sampled foliage was sent to the Environmental Analysis Laboratory (EAL) associated with Southern Cross University to test for micro- and macronutrients on a dry weight basis. pH and Electrical Conductivity were measured in a 1:5 soil: water solution. For soluble nutrients in soils, the Morgan 1 Extract was used—1:5 soil-to-solution ratio with sodium acetate (buffered to PH 4.8) (LaMotte, 2001; Wheeler and Ward, 2015). Phosphorus was measured using Bray 1, Colwell and Bray 2 methods (Rayment and Lyons, 2011). The Effective Cation Exchange Capacity was calculated as a sum of Ca, Mg, K, Na, Al and H (cmol + /kg) and soil organic carbon was estimated using the Total Carbon x 1.75 formula (Rayment and Lyons, 2011).

### Soil Compaction Measurements

To determine whether soil was compacted, the mechanical resistance of soil was measured with a hydraulic penetrometer (Meterman) with a ½ inch cone size and 600 mm shaft length. Measurements were taken a day after a rain event, when at least 15 mm fell in all study locations. Penetration resistance was recorded at 30 cm depth, 1 m away from the trunk, on four sides of each tree - south, north, west and east; these four data points were averaged for the analysis. The measurements were recorded in PSI, which were then converted into MPa.

### Data Analysis

All datasets were checked for normality with the Shapiro-Wilk test (Shapiro and Wilk, 1965) using the shapiro. test function in R 2024.09 ‘Cranberry Hibiscus’ (R Core Team, 2024). As some of the data were not normally distributed and the ‘benchmarking’ group had a lower sample size, the Kruskal-Wallis test (Kruskal and Wallis, 1952) was selected for the comparison and performed using the kruskal. test function in R (R Core Team, 2024), followed by Dunn’s test with Bonferroni correction (Dunn, 1964) for significant results. The test was conducted using the dunnTest function from the FSA package in R (Ogle et al., 2021).

Correlations among the variables were examined using the *FactoMineR* package in R and PCA was used to explore multivariate relationships among soil physicochemical properties and foliage nutrient concentrations (Lê et al., 2008); visualisations were produced using the *factoextra* package (Kassambara and Mundt, 2020).

Boxplots were created using ggplot2 (Wickham, 2016), tidyverse (Wickham et al., 2019) and ggpubr libraries (Kassambara, 2018).

## Results

### Urban Forest Assessment

**Fig. 6:**
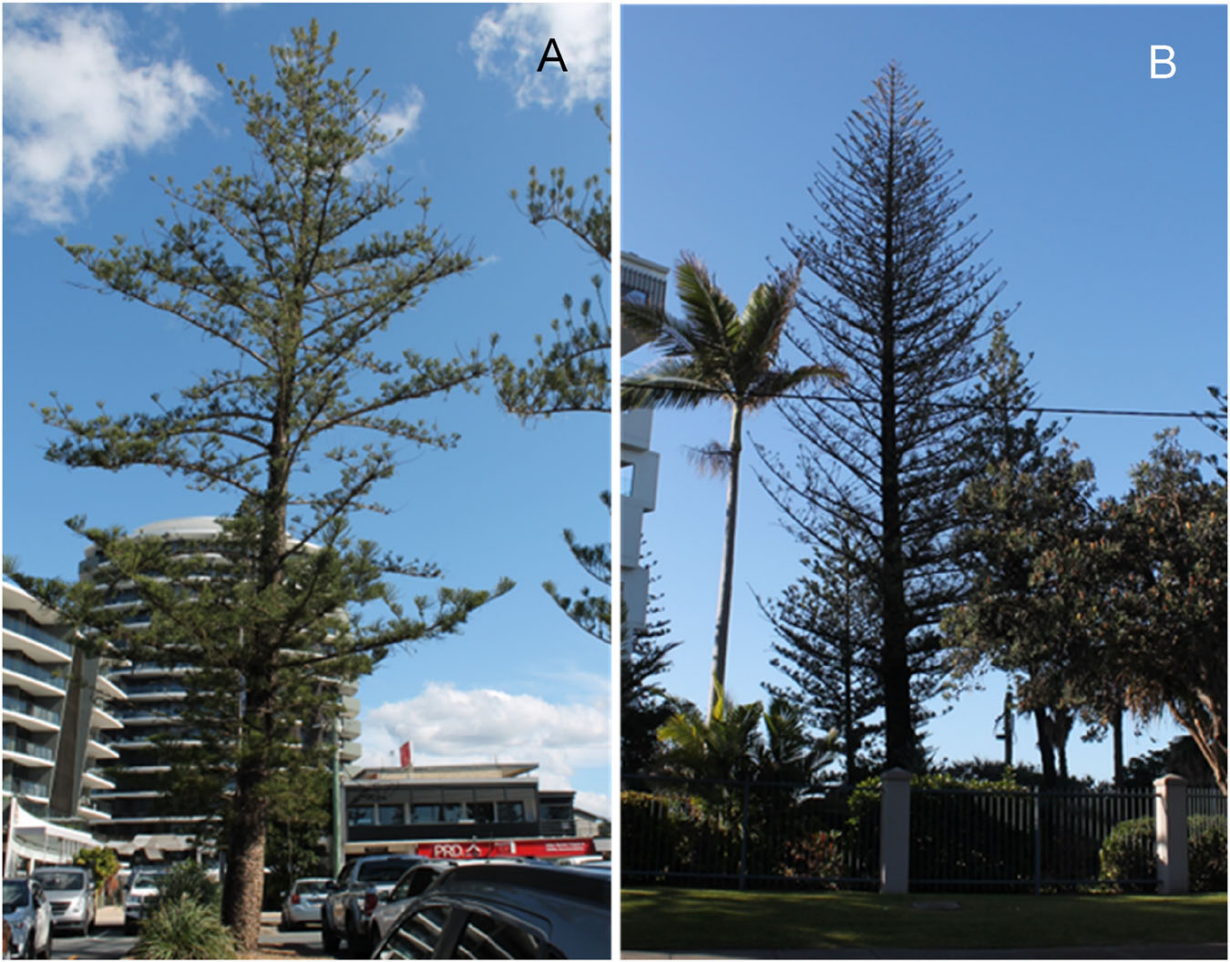
Examples of a surveyed declining tree showing over 30% decline (**A**) and an almost fully defoliated tree (**B**)

In an approximately 5-km stretch, a total of 946 *A. heterophylla* trees were visually assessed. Out of those, 149 (15.8%) showed a dieback of 30% or greater and three trees were completely denuded of foliage (Fig. 6).

### Proximity to Infrastructure and Groundcover

In the subset of trees, half (50%) in the Advanced Decline group were growing within 5 m of a carpark, while 31.3% were located near a road and a further 31.3% near a footpath. In the Moderate Decline group, fewer trees were adjacent to carparks (18.7%), a similar proportion were near roads and over two-thirds (62.5%) were located near footpaths. In contrast, none of the Benchmarking trees were found near carparks; one-third (33.3%) were adjacent to roads and half were located near footpaths (Table 2).

**Table 2:** The percentage of trees in each group located close to carparks, roads, or footpaths

	Carpark	Road	Footpath	Other
Advanced Decline	50%	31.3%	31.3%	25%
Moderate Decline	18.7%	25%	62.5%	12.5%
Benchmarking	0	33.3%	50%	16.7%

Groups include Advanced Decline (*n* = 16), Moderate Decline (*n* = 16), Benchmarking (*n* = 6) and Other (buildings, underground infrastructures, gas BBQs, shade structures, toilet blocks and playgrounds).

Turfgrass surrounded more than half of the trees (62.5%) in the Advanced Decline group. Smaller proportions were associated with bare ground and mulch with less than 1 m radius (12.5%), grass and mulch (12.5%), fallen branchlets and mulch (6.25%) and grass with fallen branchlets (6.25%). No trees in this group were recorded with mixed grass–bare ground–branchlet cover or with mulch extending to the dripline.

Greater variability in surface conditions was observed in the Moderate Decline group. The most common cover type was a combination of turfgrass and fallen branchlets (43.75%), followed by fallen branchlets with mulch with less than 1 m radius (25%) and grass alone (18.75%). Mulch extending to the dripline with branchlets was observed in 12.5% of trees, while no trees were associated solely with bare ground or grass–mulch combinations.

In the Benchmarking group, turfgrass-only and bare-ground surface cover were not observed. The most frequent conditions were mulch extending to the dripline with branchlets (33.3%) and grass with fallen branchlets (33.3%). Fallen branchlets with mulch within a 1 m radius and mixed grass–bare ground–branchlet cover each accounted for 16.7% of trees (Table 3).

**Table 3:** Types of ground cover observed in the study groups

	Advanced Decline (*n* = 16)	Moderate Decline (*n* = 16)	Benchmarking (*n* = 6)
Turfgrass	62.5%	18.75%	0%
Bare ground and mulch ( ≤ 1 m radius)	12.5%	0%	0%
Turfgrass and mulch ( ≤ 1 m radius)	12.5%	0%	0%
Fallen branchlets and mulch ( ≤ 1 m radius)	6.25%	25%	16.7%
Turfgrass and fallen branchlets	6.25%	43.75%	33.3%
Turfgrass, bare ground, branchlets	0%	0%	16.7%
Mulch (to dripline), branchlets	0%	12.5%	33.3%

Numbers represent trees attributed to each type of groundcover from each group

### Temperature

**Fig. 7:**
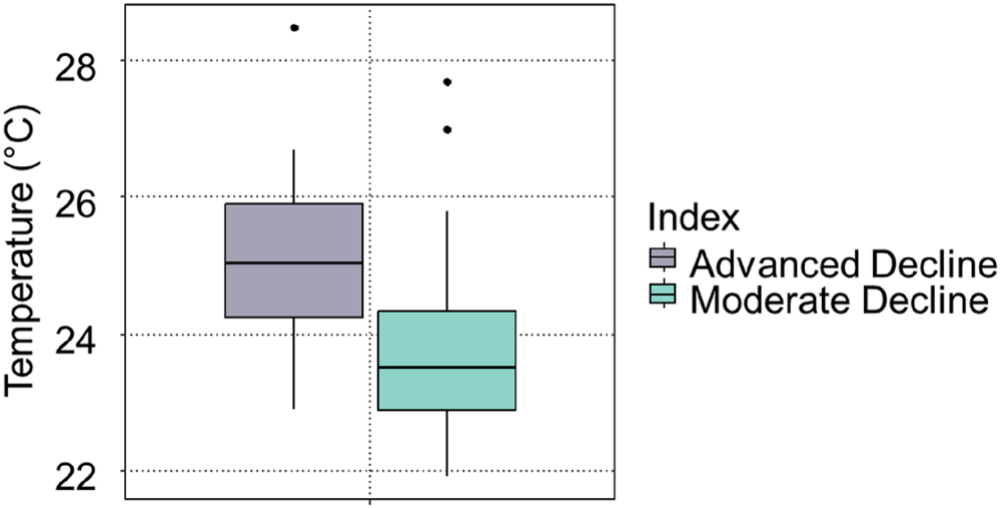
Tree trunk temperature for two groups of trees - Advanced Decline and Moderate Decline

The trunk surface temperatures ranged between 22.1 °C and 28.5 °C. There was a significant difference in the temperature of the two groups of declining trees (p < 0.05), with those in the Advanced Decline stages on average appearing to be hotter (Fig. 7).

### Soil and Foliage Nutrient Analysis

#### Soil

Trees in the Advanced Decline group were growing in loam (75%), clay loam (18.75%) and sandy soil (6.25%). The majority of the Moderate Decline trees were in loam (87.5%) and 12.5% were in clay loam soil. All Benchmarking trees were growing in sandy soil (Table 4).

**Table 4:** Soil types found in different health groups - Advanced Decline (*n* = 16), Moderate Decline (*n* = 16) and Benchmarking (*n* = 6)

	Clay loam (%)	Loam (%)	Sand (%)
Advanced Decline	18.75	75	6.25
Moderate Decline	12.5	87.5	0
Benchmarking	0	0	100

Soil pH ranged between moderately acidic (6.03) to slightly alkaline (7.20), with little difference in the soil of the Advanced Decline and Benchmarking trees, while the soil of the Moderate Decline trees had, on average, higher pH (AD 6.63 ± 0.50 SD, MD 7.15 ± 0.68 SD, B 6.51 ± 0.43 SD). Effective Cation Exchange Capacity (ECEC) was the lowest in the ‘benchmarking’ group (5.67 ± 3.32 SD) and highest in the ‘moderate decline’ group (9.79 ± 5.98 SD) (Table 5).

**Table 5:** Means ± Standard Deviation for Effective Cation Exchange Capacity for the three groups: AD Advanced Decline, MD Moderate Decline, B Benchmarking

	AD, mean ± SD (*n* = 16)	MD, mean ± SD (*n* = 16)	B, mean ± SD (*n* = 6)
ECEC (cmol + /kg)	6.50 ± 3.99	9.79 ± 5.98	5.67 ± 3.32
pH	6.63 ± 0.50	7.15 ± 0.68	6.51 ± 0.43

**Fig. 8:**
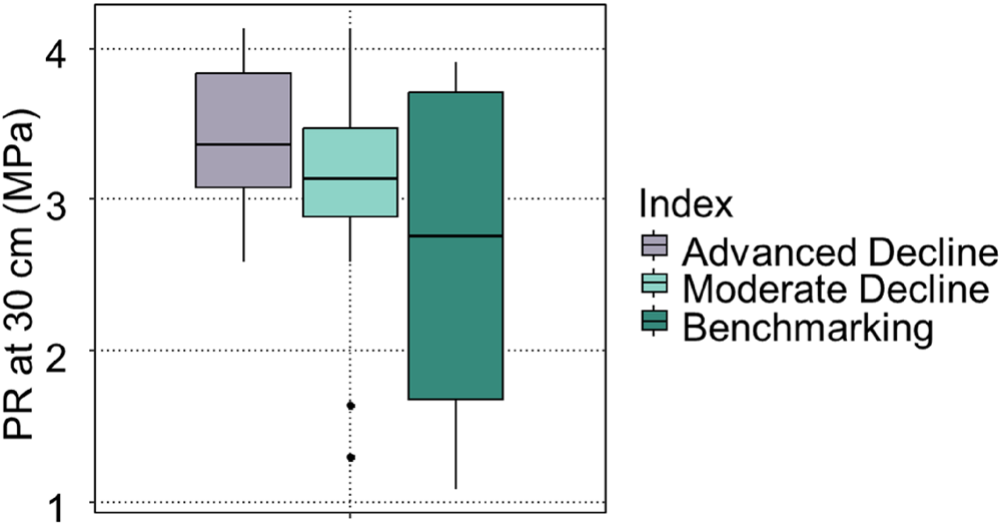
Box and whisker plots of soil penetration resistance (PR) at 30 cm depth for the three groups: Advanced Decline (*n* = 16), Moderate Decline (*n* = 16) and Benchmarking (*n* = 6), showing considerable variation among the samples and no statistical difference between the groups (*p* > 0.05)

Penetration resistance was restrictive for most of the trees (between 3 MPa and 4 MPa), although those in the Benchmarking and Moderate Decline groups had some of the trees in soil with lower levels (between 1 MPa and 3 MPa) (Fig. 8). There were no significant differences between any of the groups (*p* > 0.05); however, the soil of the trees in the Advanced Decline category was slightly more compacted on average and the Benchmarking trees had a greater range of penetration resistance.

**Fig. 9:**
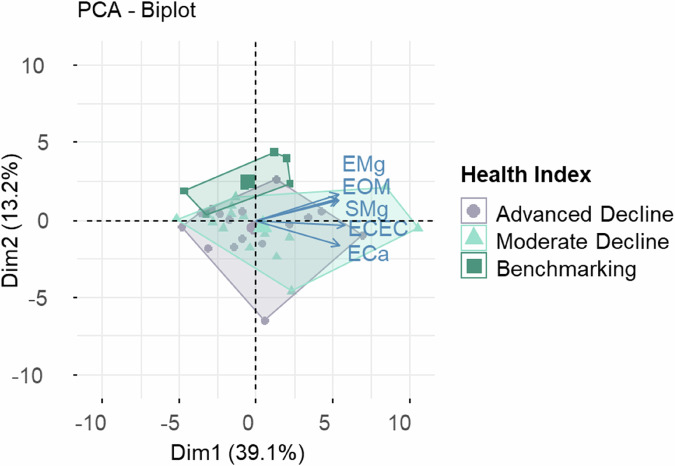
PCA plot based on the data for soil nutritional levels for the three groups - Advanced Decline (*n* = 16), Moderate Decline (*n* = 16), Benchmarking (*n* = 6) - showing the first two principal components, which explain 52.3% of the variation

Principal component analysis (PCA) showed high heterogeneity within and no clear overall distinction between the two groups of declining trees, with 52.3% of the variation explained by the first two dimensions (Fig. 9). Correlation analysis of soil variables with the principal components indicated that PC1 was primarily driven by Ca, Mg and K, while PC2 was influenced by P, Na and total N.

Collectively, the PCA demonstrated that the Advanced and Moderate Decline groups were highly heterogeneous, while Benchmarking soils were more nutrient-poor, forming a cluster somewhat different from the Decline categories.

**Fig. 10:**
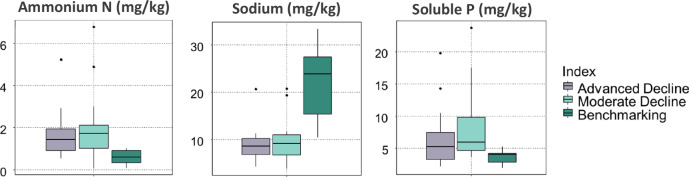
Box and whisker plots for soil sodium, ammonium-N and soluble P levels in three groups: Advanced Decline (*n* = 16), Moderate Decline (*n* = 16) and Benchmarking (*n* = 6)

A Kruskal-Wallis test followed by Dunn’s test revealed a statistically significant difference in ammonium-N between Benchmarking and Advanced Decline and Benchmarking and Moderated Deline groups (*p* = 0.04 and *p* = 0.01, respectively) and in Na for the same groups (*p* = 0.003 and *p* = 0.01, respectively). Soluble P was only significant when Moderate Decline was compared with Benchmarking trees (*p* = 0.03), while EC was significant for Advanced Decline and Benchmarking trees (*p* = 0.006) (Table 6, Fig. 10).

**Table 6:** Properties with significant statistical difference in the soil of *Araucaria heterophylla* between the studied groups—Advanced Decline (*n* = 16), Moderate Decline (*n* = 16) and Benchmarking (*n* = 6)

Nutrient	Pair	P.adj
Soluble Phosphorus (mg/kg)	Moderate Decline - Benchmarking	0.03
Ammonium Nitrogen (mg/kg)	Advanced Decline -Benchmarking	0.04
Ammonium Nitrogen (mg/kg)	Moderate Decline - Benchmarking	0.01
EC (dS/m)	Advanced Decline - Benchmarking	0.006
Sodium (%)	Advanced Decline - Benchmarking	0.003
Sodium (%)	Moderate Decline - Benchmarking	0.01

#### Trees and Foliage

Differences in tree appearance and foliage were observed among the three groups, including both physical and chemical characteristics. Resin exudation was noted in two Advanced Decline trees and one Moderate Decline tree, whereas no resin was observed on the bark of healthy trees.

Kruskal-Wallis test results for branchlet length, which ranged from 8 to 37.5 cm, indicated no statistically significant difference (*p* > 0.05), yet the branchlets of the ‘benchmarking’ trees were longer on average (Table 7).

**Table 7:** Average length of *Araucaria heterophylla* branchlets from the study groups—Advanced Decline (*n* = 16), Moderate Decline (*n* = 16) and Benchmarking (*n* = 6)

Group	Average length (cm)	Standard Deviation (cm)
Advanced Decline	19.3	7.2
Moderate Decline	21.4	8.8
Benchmarking	27.6	7.1

**Fig. 11:**
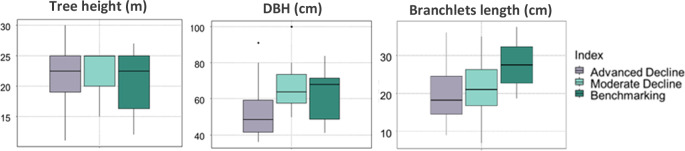
Boxplots of foliage length, trees DBH and trees height levels for the three groups: Advanced Decline (*n* = 16), Moderate Decline (*n* = 16) and Benchmarking (*n* = 6)

Analysis of DBH (diameter at breast height) data similarly did not show significant differences between groups (*p* = 0.06). Notably, there were eight younger trees, with a diameter under 50 cm, in the Advanced Decline, whereas none were found in the Moderate Decline group. Dunn’s post-hoc comparisons identified a near-significant difference between the Advanced Decline and Moderate Decline groups (*p* = 0.06). In contrast, the tree height analysis showed that the differences in tree height among the groups were less pronounced; there was no significant difference between the groups (*p* = 0.67), a finding confirmed by Dunn’s test (Fig. 11).

The first two dimensions of the PCA explained 47.8% of the variation, revealing some similarities in the nutrient composition of the foliage of ‘benchmarking’ trees and that of the two declining groups. This separation was driven by higher Na and K in the declining trees. Benchmarking trees were represented by tightly clustered points on the plot, indicating greater similarity, whereas the declining trees - both Advanced and Moderate - exhibited greater variation and overlap.

**Fig. 12:**
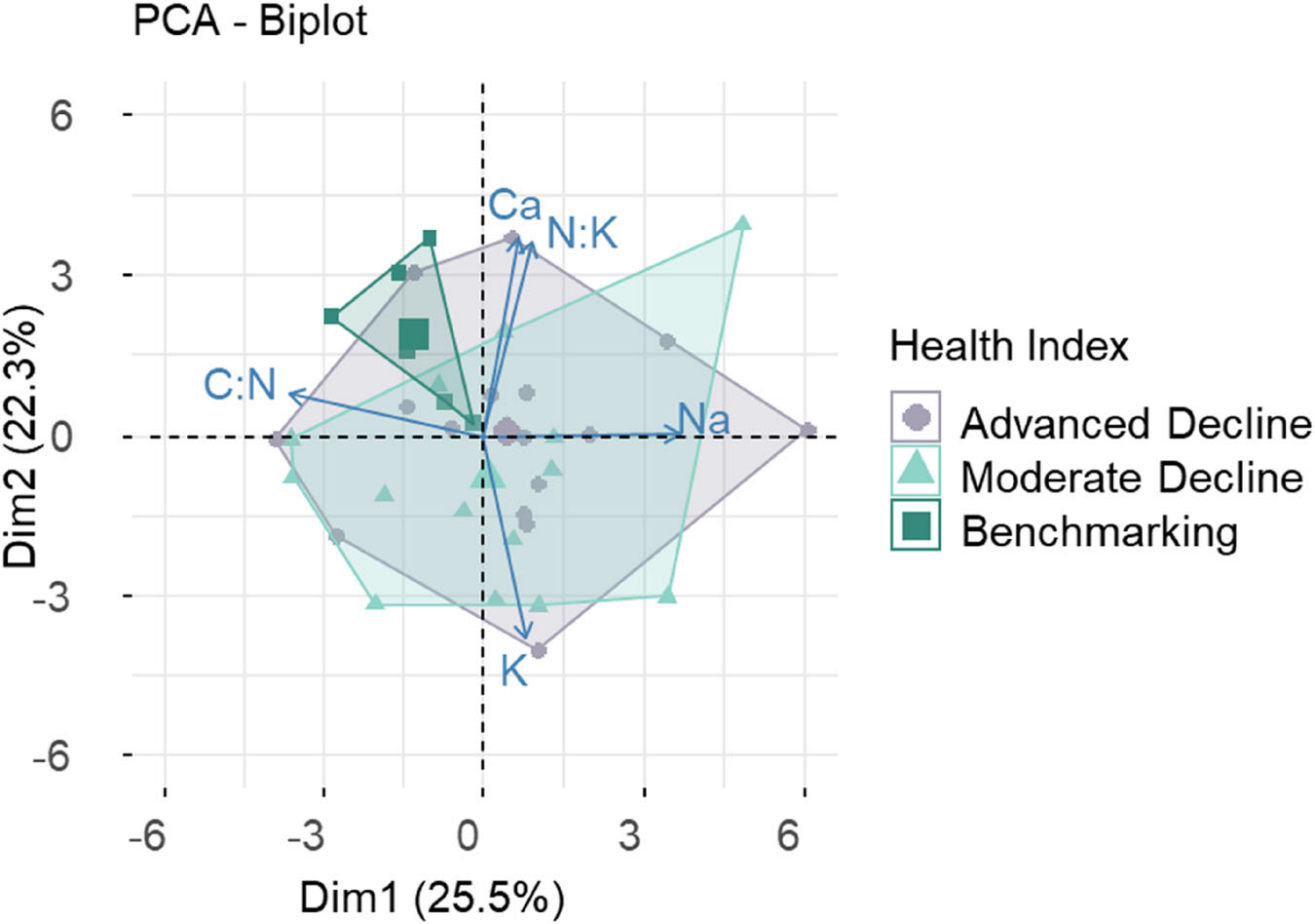
PCA plot based on the data of foliage nutritional levels for the three groups: Advanced Decline (*n* = 16), Moderate Decline (*n* = 16) and Benchmarking (*n* = 6) - showing the first two principal components, which explain 47.8% of the variation

The PCA plot (Fig. 12) of foliar nutrients revealed that the first two principal components accounted for 47.8% of the total variance. PC1 was strongly positively associated with Mg, Ca, K, total C, total N and Zn. PC2 captured variation in nutrient balance, with positive correlations with Mg, Na and Fe and negative correlations with Ca, P and pH. Trees from the Decline groups were highly heterogeneous and more dispersed along PC2. Benchmarking trees showed lower PC1 scores and more uniform PC2 values. Overall, the PCA highlighted that declining trees exhibit both elevated nutrient concentrations and greater foliar variability, whereas Benchmarking trees maintain a more similar nutrient status and have a higher C: N ratio.

On average, the Benchmarking group had the highest levels of C, Ca, Zn, Mn and B, as well as N:P ratio, N: K ratio and C: N ratio, but lowest N, P, K, Na, Cu, Fe, Co and N:S ratio (Appendix, Table 12). Yet, the difference was only significant between the groups in K, C and Mn. Potassium and Mn were lower on average in the Benchmarking trees than in the other two groups and the difference was significant when compared with the Moderate Decline group (*p* = 0.04). Carbon was significantly higher in the Benchmarking group compared with the other two (*p* = 0.0007 and *p* = 0.02) (Appendix, Table 12).

Notably, when compared to the guidelines for *A. heterophylla* presented in Reuter and Robinson (1997) Fig. 13, all groups of trees had N deficiency, Na was above the higher level in both groups of declining trees, yet within the guideline in those used for benchmarking. Zinc was just below the guideline recommendations in the Advanced Decline and Moderate Decline groups, although it just reached the low mark in the Benchmarking group. Manganese was in excess in the Advance Decline and Benchmarking trees (Appendix, Table 11; Table 8).

**Fig. 13:**
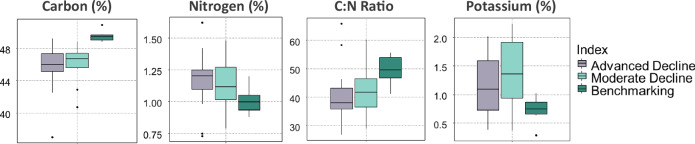
Carbon, Nitrogen, C: N Ratio and Potassium levels in foliage for the three groups: Advanced Decline (*n* = 16), Moderate Decline (*n* = 16) and Benchmarking (*n* = 6), showing statistically significant difference for both Declining and Benchmarking groups for carbon, C: N ratio, between Advanced Decline and Benchmarking for nitrogen and Moderate Decline and Benchmarking groups for potassium

**Table 8:** *P*-values for nutrients in the foliage of *Araucaria heterophylla* that demonstrated a significant statistical difference between the studied groups— Advanced Decline (*n* = 16), Moderate Decline (*n* = 16) and Benchmarking (*n* = 6)

Nutrients	Pair	P.adj
K (%)	Moderate Decline - Benchmarking	0.04
Mn (mg/kg)	Moderate Decline - Benchmarking	0.016
C (%)	Advanced Decline - Benchmarking	0.0007
C (%)	Moderate Decline - Benchmarking	0.003

Comparison of Na concentrations in soil and foliage revealed that, although soil levels were high in the Benchmarking trees, foliar concentrations remained within the recommended range. In contrast, the Declining trees had lower soil Na levels but elevated foliar Na concentrations (Table 9).

**Table 9:** Means ± Standard Deviation of Na levels in the soil and foliage for the three groups: AD - Advanced Decline, MD - Moderate Decline, B - Benchmarking

		Mean ± SD, AD	Mean ± SD, MD	Mean ± SD, B
Na (%)	Soil	9.3 ± 3.63	9.95 ± 4.54	22.11 ± 8.83
	Leaves	0.94 ± 0.58	0.87 ± 0.53	0.63 ± 0.34

## Discussion

Despite growing in parks in stands or continuously along roads, the vitality of individual *A. heterophylla* trees varied considerably, with a relatively large proportion of the species’ population in decline. This suggests it may be time to reassess management strategies in urban locations for this species.

### Weather, Climate and Urban Heat Island (UHI) Effects

Although *A. heterophylla* is an isohydric species capable of withstanding prolonged droughts, its exact levels of resilience remain unclear and knowledge about its heat tolerance is also lacking. Offord (2011) investigated the impact of high temperatures on *Araucaria* species and found that irreversible damage to *A. heterophylla’s* detached branches begins at 50.5 °C.

In urban environments, radiant heat emitted from asphalt surfaces and vehicle engines can elevate tree canopy temperatures by at least 10 °C (Offord, 2011). This can, in turn, reduce soil moisture availability and lower tree survival rates (Chen et al., 2017). On the Gold Coast, where ambient air temperatures can occasionally reach 40 °C (BoM, 2025), these heat loads may lead to irreversible physiological damage, particularly for trees situated adjacent to carparks and roads dominated by dark, heat-absorbing surfaces. In this study, the lower part of trees’ trunks was, on average, hotter in the Advanced Decline group. This can additionally be linked to lower canopy density and, hence, self-shading and to proximity to urban infrastructure - these trees were growing near carparks more often than those in Moderate Decline or healthy Benchmarking trees. This shows that while climate change is often a key focus in discussions related to urban forests, addressing UHI effects may be more pressing (Petrova et al., 2025).

In the years preceding this study, the Gold Coast experienced unusually hot and dry conditions, which likely impacted tree health, particularly in the absence of supplementary irrigation. It is reasonable to speculate that additional watering during this period could have mitigated some of the decline. As Livesley et al. (2021) note, urban land managers are increasingly recognising the impact of prolonged droughts on urban trees and are progressively implementing stormwater capture and storage systems to support irrigation and enhance tree resilience.

### Trees’ Characteristics and Maintenance

While all trees in the study groups were mature, the Advanced Decline group included a higher proportion of younger trees. It is impossible to pinpoint the cause due to the lack of historical data and monitoring, but some of the reasons could go back to the nursery production type (Wattenhofer and Johnson, 2021), weather conditions in the first years of tree growth (Nitschke et al., 2017), planting technique (Preece et al., 2023), differences in the first years’ maintenance (Roman et al., 2015), or genetic factors (Budde et al., 2016). It may also be a coincidence due to the small sample size, but it is worth further investigation.

When considering management practices, mulch application was minimal around most trees. Although there is no universal prescription for mulching, commonly cited guidelines recommend extending mulch to a radius of approximately 1.5 m from the trunk or ideally to the dripline, thereby more closely mimicking natural forest floor conditions (Maggard et al., 2012; Van Sambeek, 2016). Mulching provides multiple soil-related benefits, including moderation of soil temperature, improved moisture retention and, in some cases, increases in soil pH (Maggard et al., 2012) and nutrient availability, including total C and N levels, hot water-extractable organic C and total N, microbial biomass C and N, NO_3_^¯^ -N, total inorganic N (Hosseini Bai et al., 2014) and soil potassium (Maggard et al., 2012). In addition, mulch establishes a protective buffer that reduces the risk of mechanical injury to tree bases from lawn maintenance equipment (Morgenroth et al., 2015). Retention of fallen branchlets and foliage may further benefit trees by functioning as a form of natural groundcover.

Grass is another type of groundcover that was found under over half of the trees in the Advanced Decline group (62.5%). The percentage was lower in the Moderated Decline group (18.5%), while none of the Benchmarking trees had turfgrass as groundcover. Watson (1988) found that tree root development is influenced by the presence of turfgrass, resulting in lower root density and soil moisture. Yet, if compared to bare ground, turfgrass can offer soil stability (Braun et al., 2024) and lower ground temperature (Lindsey et al., 2025). These mixed effects suggest that groundcover type alone is unlikely to fully explain observed patterns of tree decline.

Indeed, tree location and immediate surface conditions should be interpreted within a broader ecological and management context. Factors like soil volume, genetics, the first years’ maintenance, the presence of pathogens or pests in the area and other factors can also play an important role in a tree’s growth and health (Budde et al., 2016; Consolloy, 2007; Jim, 2017; Raum et al., 2023), although these factors have not been looked at in this study. Making tree monitoring and timely data collection a good practice can fill in the knowledge gaps, guiding future tree management.

### Soil Properties and Availability of Nutrients

#### Physical Properties

Soil compaction is a well-known factor that hinders tree establishment and growth (Day and Bassuk, 1994). While moderate levels of compaction can enhance soil stability and support tree performance (Kim et al., 2022; Michael et al., 2024), excessive compaction, often resulting from heavy machinery, restricts root penetration and reduces water infiltration (Hascher and Wells, 2007). Tree roots are generally able to proliferate in soils with penetration resistance values below approximately 3 MPa (Sinnett et al., 2008).

In this study, penetration resistance values for soils surrounding most trees were high and, therefore, restrictive. However, direct comparison with standard thresholds should be interpreted with caution due to differences in penetrometer cone size. Overall, the results suggest that all trees in Advanced Decline were unlikely to have access to well-aerated soil conditions, although soils will often have cracks and voids that roots may use even when penetration resistance is high (Sinnett et al., 2008).

As measurements were limited to the upper 30 cm of the soil profile, it is not possible to determine whether compaction decreased at greater depths. Notably, soils of only some exceptionally healthy Benchmarking and Moderate Decline trees exhibited penetration resistance values below 2 MPa, with others exceeding 3 MPa. This variability suggests that compaction may differ with depth and that surface measurements alone may not fully capture root zone conditions.

All of the very healthy ‘benchmarking’ trees were found growing in sandy rudosols, which are naturally low in nutrients but drain well. Better performance of those trees may be partly attributed to the sandy soil’s porous structure, which, we can speculate, allowed deeper root penetration and possibly access to groundwater, while reducing the risk of waterlogging. At the same time, the loam and loam-clay soil in which the declining trees were growing could be less aerated and have poorer drainage, potentially hindering root function (Kozlowski, 1999). In contrast, the soil on Norfolk Island, where the trees have evolved, is well-structured, nutrient-rich and, at the same time, relatively porous (Norfolk Island Region Threatened Species Recovery Plan, 2010).

#### Nutrients

According to indicative guidelines based on concepts from ‘Albrecht’ and ‘Reams' (Albrecht, 1975; Reams, 1978), soils across all groups were low in nitrogen, particularly ammonium nitrogen. However, this did not appear to affect the health of the Benchmarking trees, which had the lowest level of the three groups, suggesting that, perhaps, mature *A. heterophylla* may require less nitrogen to thrive than very young actively growing trees.

Sodium and electrical conductivity levels were significantly higher in the soil of Benchmarking trees, yet sodium in the foliage of these trees was slightly lower on average. It appeared that healthy trees successfully facilitated sodium exclusion and restricted its uptake. The exact sodium control mechanism used by *A. heterophylla* has not been studied, but these trees likely employ a combination of strategies, which may include controlling sodium uptake, intracellular compartmentation and excretion processes (Zhang et al., 2010).

Calcium levels were the lowest in the soil of Benchmarking trees but highest in their foliage, although the difference was not significant. Calcium plays a critical role in maintaining the integrity and structure of plant cells and their walls. It regulates ion transport and selectivity, as well as other cell wall activities (Hadi and Karimi, 2012). Additionally, calcium acts as a secondary messenger during physiological and environmental stresses, such as salt stress and in other cellular processes (Pathak et al., 2021). During salt stress, a cytosolic calcium signal activates the SOS3 protein, which initiates a series of processes leading to the efflux of Na⁺ (Hadi and Karimi, 2012). The reasons why calcium was taken up by some plants better than by others need to be investigated further, but it is possible that the root system of healthy trees is better developed due to a more porous soil structure.

Trees in urban areas reduce atmospheric pollution and fix atmospheric CO_2_ (Fares et al., 2017). Benchmarking trees had consistently high foliage carbon concentration and were potentially more efficient at fixing carbon dioxide into their biomass than the declining trees. This could be due to better water availability and, therefore, unconstrained productivity (Liu et al., 2017), as carbon sequestration is known to be affected during drought (Martínez-Sancho et al., 2022). Approximately half of the declining trees were found near carparks and roads. As vehicular pollutants can clog the stomata of trees and affect transpiration, this could also result in lower carbon fixation and, therefore, tree growth (Manikandan et al., 2021).

Beyond these differences, there were no significant variations in nutrient load between the groups, suggesting that nutrition was not the only determinant of tree success or failure.

### Planning for the Future

It is challenging to identify a single factor responsible for the decline of *A. heterophylla*, as multiple issues likely contribute to its susceptibility to external stresses. A large portion of trees planted near roads and car parks exhibited poor health, suggesting that this species may struggle to tolerate the vehicular pollution or high temperatures often observed in summer near tarmac surfaces. Additionally, impermeable surfaces and soil compaction in these areas can reduce water absorption, creating dry environments that further stress the trees and make them vulnerable to biotic stressors.

Some of these conditions—compaction, drought and heat—may lead to poorly developed root systems. However, as this hypothesis has not been tested, it remains speculative. Compounding these challenges is a general lack of data and consistent tree monitoring, which is a common issue in large cities with extensive urban forests. This lack of information makes it more difficult to evaluate and understand the processes contributing to tree decline.

While the data collected on soil and plant nutritional values could not confirm that the lack of one nutrient or another plays a dramatic role in the health of *A. heterophylla*, it is good to remember that adequate nutrient levels not only result in healthier trees but also can improve their resilience and help to control plant diseases (Dordas, 2008). On the other hand, nutrient excess has to be monitored and, when possible, avoided.

Based on these findings, the following steps are suggested to support the health of *A. heterophylla*:

1. Avoid planting this species near roads, car parks and other areas with high heat and compaction risk.

2. Consider supplemental irrigation during periods of inadequate rainfall to mitigate the effects of drought and compaction.

3. Mulch to dripline is required to ensure root protection, temperature regulation and the improvement of soil moisture and soil health.

4. Implement a consistent program for monitoring tree health and recording data. This will enhance understanding of the factors contributing to decline and enable quicker diagnosis and intervention for issues like dieback.

By addressing these key factors, it may be possible to reduce the species’ vulnerability and ensure its long-term survival in urban environments.

### Study Limitations

This study highlights abiotic factors that could be important in informing future management, yet are insufficient to determine causation. Working with existing tree plantations and the realities of urban forest management meant there were some limitations to experimental design.

This study was limited to one region and while urban planning strategies generally share common features and constraints across cities and even countries, variations in climate and management may lead to different outcomes.

Out of 38 trees assessed, only six could be classified as in excellent health. These trees were situated in sandy soils, which raised uncertainty as to whether the apparent differences were due to genuinely more suitable soil conditions for the species or simply the effect of the small sample size.

Additionally, because the trees were located in high-profile parks and the soil results had already been analysed by a third party, the data were not provided in full detail. Only three soil texture classes were reported—sand, loam and clay loam— without accompanying percentage distributions. As the soil samples were collected and processed by an external organisation, only limited methodological information was available. Although it is known that the samples were homogenised prior to analysis, the number of cores collected per sample is unknown.

Finally, groundwater data were not available for the area where the study took place, which limited the ability to assess potential subsurface water influences on tree health and soil conditions.

This study did not examine biotic threats to *A. heterophylla*, which have been identified in previous research as potential contributors to decline. Fungal pathogens affecting this species are documented in a separate study.

## Conclusion

The study investigated abiotic factors contributing to the decline of *A. heterophylla* on the Gold Coast. Findings suggest that urban infrastructure, extreme weather events and some soil properties are likely underlying but not direct causes of the observed decline. Management recommendations include increasing planting distance from hard infrastructure, improving soil conditions, implementing irrigation systems and conducting regular health monitoring. Abiotic stressors appear to weaken trees, reducing their vigour and potentially increasing their susceptibility to pests and pathogens.

## Data Availability

No datasets were generated or analysed during the current study.

## Appendix

Table 10

**Table 10:** Mean and Highest Mean monthly temperatures for the two locations for all years recorded and the temperature difference between the two locations

Month	Coolangatta Mean (°C)	NI Mean (°C)	Difference (°C)	Coolangatta Highest Mean (°C)	NI Highest Mean (°C)	Difference (°C)
Jan	28.5	24.6	3.9	30.1	26.1	4
Feb	28.4	25	3.4	30.6	27.1	3.5
Mar	27.5	24.4	3.1	29	25.9	3.1
Apr	25.6	22.8	2.8	27.1	24.5	2.6
May	23.2	21	2.2	25.1	22.3	2.8
Jun	21.1	19.4	1.7	22.3	20.6	1.7
Jul	20.7	18.4	2.3	22	19.9	2.1
Aug	21.7	18.4	3.3	24	19.6	4.4
Sep	23.5	19.1	4.4	25.6	20.7	4.9
Oct	24.7	20.3	4.4	26.6	22.3	4.3
Nov	26.1	21.8	4.3	27.7	24.1	3.6
Dec	27.5	23.5	4	29.7	25.4	4.3

NI - Norfolk Island. Data obtained from BOM (2025)

Table 11

**Table 11:** Means ± Standard Deviation of soil nutrient levels for the three groups: AD Advanced Decline, MD Moderate Decline, B Benchmarking

	AD, mean ± SD (*n* = 16)	MD, mean ± SD (*n* = 16)	B, mean ± SD (*n* = 6)
Soluble Ca (mg/kg)	428.13 ± 230.67	867.23 ± 737.26	313.39 ± 162.32
Soluble Mg (mg/kg)	194.30 ± 129.49	249.36 ± 160.66	162.94 ± 84.28
Soluble K (mg/kg)	74.14 ± 47.27	91.81 ± 40.38	60.54 ± 29.34
Soluble P (mg/kg)	5.62 ± 3.56	9.10 ± 5.93	3.86 ± 1.10
P (mg/kg)	34.15 ± 50.09	26.00 ± 27.37	12.15 ± 6.50
Nitrate N (mg/kg)	4.82 ± 5.00	4.58 ± 3.66	3.71 ± 3.29
Ammonium N (mg/kg)	1.57 ± 0.63	1.97 ± 1.70	1.15 ± 1.54
Exchangeable Ca (mg/kg)	725.80 ± 453.76	1256.18 ± 830.96	531.46 ± 346.42
Exchangeable Mg (mg/kg)	233.15 ± 160.81	289.29 ± 212.87	201.70 ± 123.43
Exchangeable K (mg/kg)	106.29 ± 74.66	120.43 ± 62.05	74.97 ± 35.99
Exchangeable Na (mg/kg)	144.98 ± 112.36	179.84 ± 75.29	269.78 ± 230.06
Exchangeable Al (mg/kg)	2.47 ± 1.02	3.11 ± 1.28	1.98 ± 0.86
Exchangeable H (mg/kg)	1.05 ± 0.10	1.03 ± 0.09	1.00 ± 0.00
ECEC (cmol + /kg)	6.50 ± 3.99	9.79 ± 5.98	5.67 ± 3.32
Calcium (%)	56.01 ± 8.57	62.12 ± 9.01	46.19 ± 11.61
Potassium (%)	3.84 ± 1.41	3.28 ± 1.02	2.96 ± 1.01
Hydrogen (%)	1.38 ± 2.12	0.28 ± 0.80	0.01 ± 0.03
Total Carbon (%)	2.03 ± 1.08	2.41 ± 1.62	1.75 ± 1.31
Total Nitrogen (%)	0.15 ± 0.09	0.17 ± 0.10	0.10 ± 0.06
Ca: Mg Ratio	2.09 ± 0.75	3.12 ± 2.56	1.60 ± 0.54
Magnesium (%)	28.89 ± 6.20	24.81 ± 7.31	29.75 ± 3.64
Zn (mg/kg)	6.23 ± 4.30	9.86 ± 5.11	6.58 ± 7.47
Fe (mg/kg)	105.39 ± 54.02	83.82 ± 38.32	110.69 ± 62.95
B (mg/kg)	0.60 ± 0.36	0.66 ± 0.31	0.59 ± 0.39
Si (mg/kg)	14.35 ± 7.50	23.14 ± 14.29	14.98 ± 7.49
Aluminum (%)	0.55 ± 0.40	0.44 ± 0.29	0.64 ± 0.65
C: N Ratio	13.75 ± 3.89	14.07 ± 3.10	14.53 ± 6.06
pH	6.63 ± 0.50	7.15 ± 0.68	6.51 ± 0.43
S (mg/kg)	6.75 ± 4.39	16.35 ± 15.48	14.21 ± 12.82

Table 12

**Table 12:** Means and standard deviations of nutrients of the foliage of Araucaria heterophylla

Index	Advanced Decline ± SD (*n* = 16)	Moderate Decline ± SD (*n* = 16)	Benchmarking ± SD (*n* = 16)	Indicative Guideline
N %	1.17 ± 0.23	1.13 ± 0.19	1.01 ± 0.11	1.4-2.6
P %	0.19 ± 0.04	0.19 ± 0.07	0.17 ± 0.06	0.16-0.3
**K %**	**1.13** ± **0.52**	**1.37** ± **0.57**	**0.73** ± **0.25**	**1-1.8**
S %	0.23 ± 0.06	0.23 ± 0.08	0.23 ± 0.04	
**C %**	**45.63** ± **2.84**	**46.3** ± **2.6**	**49.55** ± **0.75**	
Ca %	1.56 ± 0.38	1.43 ± 0.42	1.78 ± 0.23	1.4-2
Mg %	0.49 ± 0.14	0.4 ± 0.13	0.49 ± 0.07	0.3-0.5
Na %	0.94 ± 0.58	0.87 ± 0.53	0.63 ± 0.34	0.15-0.8
Cu (mg/kg)	6.47 ± 2.18	6.6 ± 2.59	4.52 ± 1.42	
Zn (mg/kg)	33.77 ± 13.12	29.18 ± 9.87	35.96 ± 11.37	35-80
**Mn (mg/kg)**	**89.11** ± **64.30**	**53.07** ± **28.02**	**144.95** ± **80.04**	**10-70**
Fe (mg/kg)	220.04 ± 190.40	202.97 ± 193.08	104.93 ± 28.84	100-400
B (mg/kg)	15.51 ± 6.98	16.73 ± 3.92	18.24 ± 4.52	12-30
Co (mg/kg)	0.44 ± 0.22	0.37 ± 0.30	0.35 ± 0.10	
N:S ratio	5.37 ± 0.96	5.2 ± 1.47	4.59 ± 0.81	
N:P ratio	6.58 ± 1.73	6.41 ± 2.01	6.73 ± 2.31	
N:K ratio	1.3 ± 0.72	1.05 ± 0.80	1.59 ± 0.72	
C:N ratio	40.65 ± 9.93	42.43 ± 8.38	49.62 ± 5.52	

Nutrients with statistically significant difference shown in bold, *p*-values provided for groups (MD–Moderated Decline, AD–Advanced Decline, B–Benchmarking). Indicative guideline is extracted from Reuter and Robinson (1997)
